# Tumor Suppressor p53 Down-Regulates Programmed Cell Death Protein 4 (PDCD4) Expression

**DOI:** 10.3390/curroncol30020124

**Published:** 2023-01-27

**Authors:** William H. Yang, Andrew P. George, Chiung-Min Wang, Richard H. Yang, Avery M. Duncan, Darshti Patel, Zachery D. Neil, Wei-Hsiung Yang

**Affiliations:** Department of Biomedical Sciences, Mercer University School of Medicine, Savannah, GA 31404, USA

**Keywords:** p53, PDCD4, transcriptional activity

## Abstract

The programmed cell death protein 4 (PDCD4), a well-known tumor suppressor, inhibits translation initiation and cap-dependent translation by inhibiting the helicase activity of EIF4A. The EIF4A tends to target mRNAs with a structured 5′-UTR. In addition, PDCD4 can also prevent tumorigenesis by inhibiting tumor promoter-induced neoplastic transformation, and studies indicate that PDCD4 binding to certain mRNAs inhibits those mRNAs’ translation. A previous study demonstrated that PDCD4 inhibits the translation of p53 mRNA and that treatment with DNA-damaging agents down-regulates PDCD4 expression but activates p53 expression. The study further demonstrated that treatment with DNA-damaging agents resulted in the downregulation of PDCD4 expression and an increase in p53 expression, suggesting a potential mechanism by which p53 regulates the expression of PDCD4. However, whether p53 directly regulates PDCD4 remains unknown. Herein, we demonstrate for the first time that p53 regulates PDCD4 expression. Firstly, we found that overexpression of p53 in p53-null cells (H1299 and Saos2 cells) decreased the PDCD4 protein level. Secondly, p53 decreased *PDCD4* promoter activity in gene reporter assays. Moreover, we demonstrated that mutations in p53 (R273H: contact hotspot mutation, and R175H: conformational hotspot mutation) abolished p53-mediated PDCD4 repression. Furthermore, mutations in the DNA-binding domain, but not in the C-terminal regulatory domain, of p53 disrupted p53-mediated PDCD4 repression. Finally, the C-terminal regulatory domain truncation study showed that the region between aa374 and aa370 is critical for p53-mediated PDCD4 repression. Taken together, our results suggest that p53 functions as a novel regulator of PDCD4, and the relationship between p53 and PDCD4 may be involved in tumor development and progression.

## 1. Introduction

Cancer is one of the most dreaded and dangerous diseases of the 21st century for humans. Currently, the human cancer mortality rate is approximately 11–25%, according to Cancer Statistics on the NCI website (https://www.cancer.gov/about-cancer/understanding/statistics, 25 September 2020). The p53 gene, the 1993 Molecule of the Year, is a significant cellular regulator in human cancers, and its important role in response to DNA damage has been highlighted by the discovery that more than 50% of human cancers harbor p53 mutations [1]. In addition to mutations, the other major mechanism leading to the dysfunction of the *TP53* gene is the downregulation of wild-type (WT) p53 by MDM2/MDM4 [2]. Throughout decades of studies, hundreds of p53 target genes have been identified so far, including *p21*, *PUMA*, *GADD45*, etc. [3,4,5]. As a transcription factor and tumor suppressor, p53 is regulated by several post-translational modifications, such as phosphorylation [6], acetylation [7], methylation [8], and SUMOylation [9], especially in the C-terminal regulatory domain. Recently, p53-dependent ferroptosis, an iron-dependent form of non-apoptotic cell death, has been linked to p53-mediated tumor suppression [10]. Overall, as the most studied protein, p53 regulates DNA repair [11], apoptosis [12], metabolism [13], autophagy [14], translation control [15], cell cycle arrest [16], and feedback mechanisms including the MDM2-p53 pathway [17]. 

The programmed cell death protein 4 (PDCD4), a well-known tumor suppressor, inhibits translation initiation and cap-dependent translation by inhibiting the helicase activity of EIF4A [18]. The EIF4A tends to target mRNAs with a structured 5′-UTR [19]. In addition, PDCD4 can also prevent tumorigenesis by inhibiting tumor promoter-induced neoplastic transformation [20], and studies indicate that PDCD4 may inhibit the translation of certain mRNAs via direct binding [21]. A previous study demonstrated that PDCD4 inhibits the translation of p53 mRNA [22], whose protein is a key regulator of the cell cycle and cell death. The inhibition of p53 is via an EIF4A-dependent mechanism. The study further demonstrated that treatment with DNA-damaging agents resulted in the downregulation of PDCD4 expression and an increase in p53 expression. The results suggest a potential mechanism by which p53 regulates the expression of PDCD4. Along with p53, PDCD4 inhibits the translation of many other genes (*c-Myb*, *Bcl-x_L_*, *XIAP*, *Sin1*, etc.) to prevent tumorigenesis [21,23,24,25]. Furthermore, the PDCD4 is highly conserved among vertebrates and is frequently downregulated in multiple different types of cancer, including colon [26], liver [27], breast [28], lung [29], pancreas [30], and more. However, whether p53 directly regulates PDCD4 remains unknown. Therefore, in this study, we assessed the role of p53 in regulating the expression of PDCD4. (Note: Part of the current study was previously presented at the American Association for Cancer Research (AACR) annual meeting in 2021 and has been reprinted/adapted with permission from Ref. [31], 2021, AACR).

## 2. Materials and Methods

### 2.1. Chemicals and Reagents

Both cell culture medium and cell culture reagents were purchased from Thermo Fisher Scientific (Waltham, MA, USA). Antibodies against p53, PDCD4, FOXP2, YB-1, and β-Actin were purchased from Santa Cruz Biotechnology Inc. (Santa Cruz, CA, USA). Antibodies against Caspase 3 and ERK1/2 were purchased from Cell Signaling Technology Inc. (Danvers, MA, USA). P53 siRNA (h) was purchased from Santa Cruz Biotechnology Inc. (Santa Cruz, CA, USA). Luciferase activity was measured using the Dual-Luciferase Reporter Assay System (Promega, Madison, WI, USA).

### 2.2. DNA Constructs

Human *p53*-pcDNA4 expression plasmid (with HIS tag) was generated by RT-PCR in the Yang lab. Two primers (5′- GGATCCACTAGTCatggaggagccgcag -3′ and 5′- CGGGCCCTCTAGACTCGAG tcagtctgagtcag -3′) were used to synthesize the human *p53* fragment, which was then cloned into the expression vector pcDNA4. Human *p53* mutant and truncated expression plasmids used in this study were created by PCR-based In-Fusion technology from WT *p53*-pcDNA4 expression plasmids (In-Fusion HD Cloning Kit, Takara Bio, San Jose, CA, USA). The human *PDCD4* promoter (−242/+355 bp) Pgl4 plasmid was generated by PCR in the Yang lab. Two primers (5′- GAATGGGTGAGCTGGGTCGAGGAAGCATCATCCTCGTCCCCATC -3′ and 5′- AAGCTTTGCGGGGCTACAAGAAG -3′) were used to amplify the human *PDCD4* promoter fragment. Plasmids containing the human PDCD4 promoter with the p53 response element (RE) mutant were created by PCR-based mutagenesis (QuikChange Lightning site-directed mutagenesis kit, Agilent/Strategene, La Jolla, CA, USA). All DNA plasmid constructs were verified by Sanger nucleotide sequencing.

### 2.3. Cell Culture and Transfection

The cell culture and transfection methods were carried out as previously described [32]. H1299 (CRL-5803), MCF7 (HTB-22), and Saos2 (HTB-85) cells were purchased from the American Type Culture Collection (Manassas, VA, USA). The cells were grown/maintained in Dulbecco’s modified Eagle medium (DMEM) with fetal bovine serum (10% for H1299 cells and 15% for Saos2 cells) and 1% Pen/Strep antibiotics (GIBCO/Life Technologies, Grand Island, NY, USA) in a humidified incubator (5% CO_2_ at 37 ℃) and cultured for less than six months. Both cells were also routinely checked and confirmed negative for Mycoplasma contamination using a PCR detection kit (Millipore-Sigma, Burlington, MA, USA) throughout the study. After incubation, the cells were transfected with specific expression plasmids described in each assay using the Fugene HD Transfection Reagent (Roche, Madison, WI, USA). Forty-eight hours after transfection, the cells were harvested and lysed for use in promoter luciferase reporter assays or Western blot analysis. 

### 2.4. PDCD4 Promoter Luciferase Reporter Assays

The reporter assays were carried out as previously described [32]. Cells were cultured in 24-well plates overnight and then transiently transfected with the *PDCD4* promoter-firefly luciferase plasmid, the internal control pRL-TK plasmid (which encodes *Renilla* luciferase activity), and with or without *p53* WT or mutant expression plasmids in the presence of Fugene HD Transfection Reagent (Roche, Madison, WI, USA). Forty-eight hours after transfection, the cells were harvested and lysed in a passive lysis buffer (Promega, Madison, WI, USA). Luminescence was detected with the Dual-Luciferase Reporter Assay System Kit (catalog no. E1960, Promega, Madison, WI, USA) using a luminometer (Turner Designs, Sunnyvale, CA) according to the manufacturer’s instructions. The firefly luciferase activity was normalized by calculating the ratio to *Renilla* luciferase activity. The relative luciferase activity was calculated as a fold change compared to the control groups. All experiments were performed three times in a triplicate setting.

### 2.5. Western Blot Analysis

The Western blot analysis was performed as previously described [32]. After 48 hours of transfection, cells were washed with ice-cold PBS and lysed with ice-cold 1X RIPA buffer supplemented with phosphatase inhibitors and protease inhibitors. The protein contents of the high-speed supernatant were determined using the BCA^TM^ Protein Assay Kit assay (Pierced/Thermo Scientific, Rockford, IL, USA). Equal amounts of protein (approximately 40 µg) were resolved on 8–10% polyacrylamide-SDS gels and transferred to polyvinylidene difluoride (PVDF) membrane (Bio-Rad, Hercules, CA, USA) by wet electrophoretic transfer. The membranes were blocked with 5% nonfat milk and probed with specific primary antibodies first and then with specific secondary antibodies. Antibodies were diluted in 5% dry milk powder in TBST (0.1% Tween-20/TBS) buffer. Blots were visualized using the Supersignal West Dura Extended Duration Substrate kit (Pierce Chemical Co., Rockford, IL, USA). The intensity of the protein band was quantified by the ImageJ software (NIH, Bethesda, MD, USA).

### 2.6. Statistical Analysis

Statistical analysis and comparisons were performed using the Student’s *t*-test to determine the statistical significance between groups. A value of *p* < 0.05 was considered statistically significant between groups.

## 3. Results

### p53 Decreases PDCD4 Protein Level

**Figure 1:**
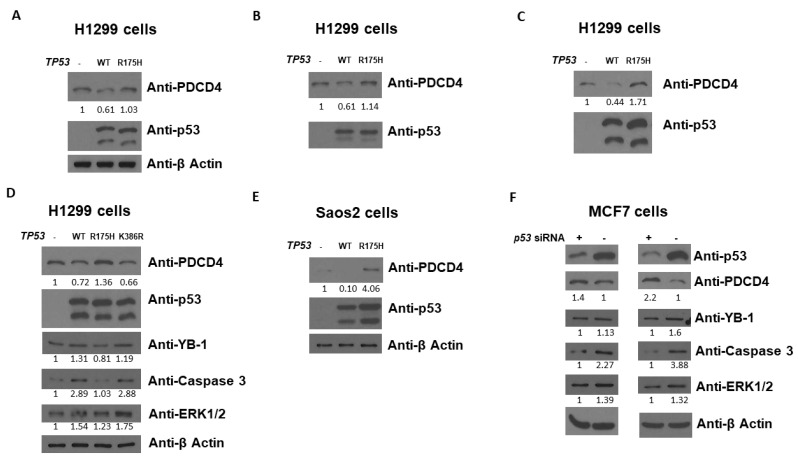
p53 decreases PDCD4 protein level. Western blot analysis of PDCD4 expression from H1299 (**A**–**D**) and Saos2 (**E**) cells transfected without or with WT or mutant *p53* expression plasmids. (**F**) Western blot analysis of PDCD4 expression from MCF7 cells treated with p53 siRNA. The expression levels of p53 and PDCD4 were determined using anti-p53 and anti-PDCD4 immunoblotting, respectively. The expression levels of YB-1, Caspase 3, and ERK1/2 were also determined on panel (**D**,**F**). The β-Actin levels were determined for equal loading on panel (**A**,**D**–**F**). The original WB can be found in Supplementary Material.

In order to dissect the relationship between p53 and PDCD4, we first investigated the role of p53 in PDCD4 expression. We used H1299 (human lung epithelial cancer) and Saos2 (human osteosarcoma) cells to evaluate whether p53 affects PDCD4 protein expression since both cells express little to no endogenous p53. As shown in Figure 1A–D using H1299 cells, WT p53 induction by transient transfection decreased the expression levels of PDCD4. However, in R175H (a conformation hotspot mutant), p53 induction did not reduce PDCD4 expression, suggesting that disruption of p53 3D conformation and DNA binding activity leads to a loss of p53-mediated PDCD4 repression. A similar result was observed on Saos2 cells, as shown in Figure 1E. To confirm the results of Figure 1A-1E, we next knockdown p53 by the siRNA system in MCF7 cells (which express endogenous WT p53) to evaluate whether p53 affects PDCD4 expression. As shown in Figure 1F, the reduction of p53 by siRNA increased the expression levels of PDCD4. Several PDCD4 downstream targets have been reported, including YB-1 [33], Caspase 3 [34], and ERK1/2 [35] in different pathways. As shown in Figure 1D,F, the caspase 3 pathway seems the most important in the p53-PDCD4 axis. Overall, these findings indicate that p53 has the potential to down-regulate PDCD4 expression, and the p53-PDCD4-Caspase 3 axis likely is involved in the apoptosis pathway triggered by p53.

**Figure 2:**
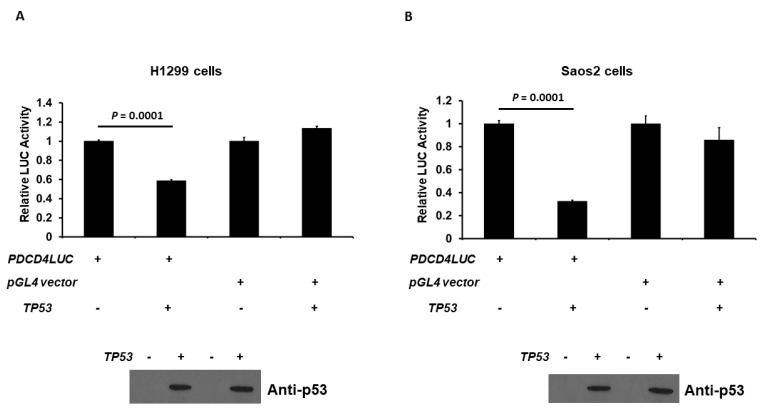
p53 down-regulates *PDCD4* transcription. p53 reduces *PDCD4* promoter activity in H1299 (**A**) and Saos2 (**B**) cells. Cells in a 24-well plate were co-transfected with either *PDCD4* luciferase reporter plasmid or pGL4 empty vector and *pRL-TK Renilla* control vector, with or without p53 expression plasmids, by Fugene HD. After forty-eight hours of transfection, luciferase activities were analyzed by the Dual-Luciferase Reporter System and normalized to the control *Renilla* activity. Relative LUC activity was calculated and plotted. The protein levels of p53 in the cells from the reporter assays were confirmed using anti-p53 immunoblotting. The original WB can be found in Supplementary Material.

As p53 decreases PDCD4 protein expression, as shown in Figure 1, we next examined the effect of human p53 on *PDCD4* promoter activation. The *PDCD4* promoter (−242/+355 bp)-LUC reporter plasmid was co-transfected with the *p53* expression plasmid into H1299 or Saos2 cells, and *PDCD4* promoter activity was measured by quantifying the LUC activity in cell lysates 48 h after transfection. As shown in Figure 2A, the expression of p53 generated a decrease in the promoter activity of *PDCD4* gene transcription in H1299 cells. As expected, p53 did not repress the pGL4 vector, which does not contain the PDCD4 promoter. Similar results were observed in Saos2 cells in Figure 2B. This finding indicates that p53 is a repressor of *PDCD4* transcription independent of cell types.

**Figure 3:**
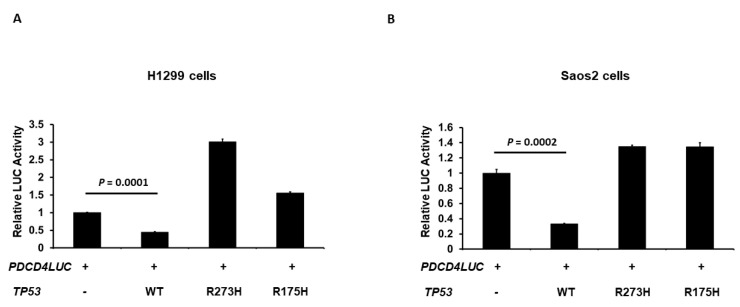
Hot-spot mutations on p53 abolish p53’s ability to down-regulate *PDCD4* transcription. H1299 (**A**) and Saos2 (**B**) cells in a 24-well plate were co-transfected with the *PDCD4* luciferase reporter plasmid and *pRL-TK Renilla* control vector and either WT, R273H, or R175H *p53* expression plasmids by Fugene HD. After forty-eight hours of transfection, luciferase activities were analyzed by the Dual-Luciferase Reporter System and normalized to the control *Renilla* activity. Relative LUC activity was calculated and plotted.

In order to further confirm the result from Figure 1 and Figure 2, we next examined the effect of the human p53 hotspot mutations of cancer on the transcriptional activity of the *PDCD4* promoter. H1299 cells were co-transfected with the *PDCD4* promoter-LUC reporter plasmid and with either wild-type (WT), R273H (contact hotspot mutation), or R175H (a conformational hotspot mutation) *p53* expression plasmid. As shown in Figure 3A, in H1299 cells, while the WT p53 repressed *PDCD4* promoter activity as expected, the R273H and R175H p53 did not reduce *PDCD4* promoter activity. Similar results were observed in Saos2 cells in Figure 3B, which suggests that proper DNA contact and structural conformation are critical for p53-mediated PDCD4 repression. 

**Figure 4:**
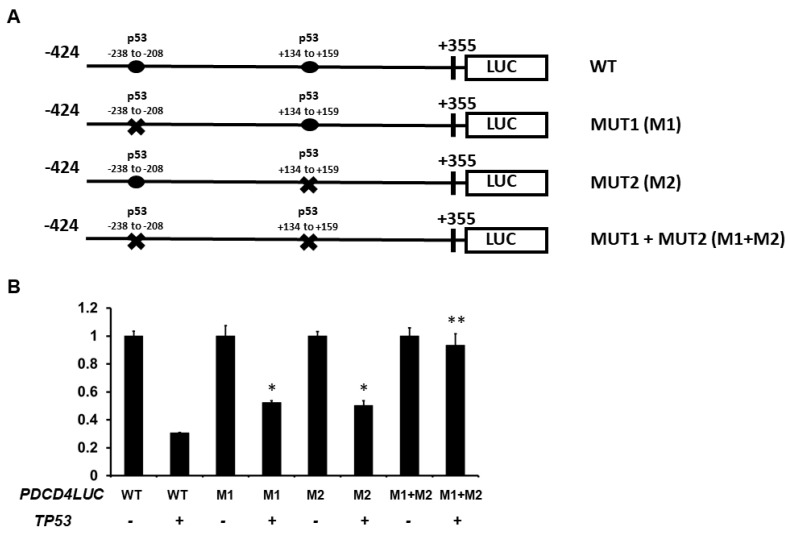
Regions of the *PDCD4* promoter important for transcriptional down-regulation by p53. (**A**) Experimental design: H1299 cells were co-transfected with *p53* expression plasmids with either −424 wild-type (WT), −238 to −208 RE mutated (M1), +134 to +159 RE mutated (M2), or both M1 + M2 *PDCD4* promoter constructs. (**B**) Reporter Assays: After forty-eight hours of transfection, luciferase activities were analyzed by the Dual-Luciferase Reporter System and normalized to the control *Renilla* activity. Relative LUC activity was calculated and plotted. * indicates a *p*-value < 0.05 compared with *PDCD4*LUC WT + *TP53*. ** indicates a *p*-value < 0.01 compared with *PDCD4*LUC WT + *TP53*.

Additionally, to determine whether the p53 response elements (REs) are located and required for p53-mediated PDCD4 expression, we first searched for potential p53 binding site(s) on the PDCD4 promoter region. We identified two potential p53 binding sites in the human PDCD4 promoter regions based on the known repressing p53 RE sequence: RRXCXXGXYX-XRXCXXGXYY (X is A/T/C/G). Two potential p53 binding sites are located −238 to −208 bp upstream and +134 to +159 bp downstream of the transcription start site, which suggests that p53 may directly regulate PDCD4 transcription (Figure 4A). We next generated −238 to −208 bp mutant (GAGCTGGGTCGaggaagcttcATCCTCGTCC -> GAGAAAAGTCGaggaagcttcATCAAAATCC) (M1), +134 to +159 bp mutant (GGGGCCGGCTGaccagGAACCTGGGC -> GGGGAAAACTGacccagGAAAAAAGGGC) (M2), and a M1+M2 mutant that contains both M1 and M2 mutations. As shown in Figure 4B, either M1 or M2 mutations resulted in approximately 50% loss of p53-mediated *PDCD4* promoter repression. Notably, mutations in both M1 and M2 dramatically reduced *PDCD4* promoter activity (approximately 90% loss). Together, these results indicate that both REs are involved in p53-mediated *PDCD4* repression. 

**Figure 5:**
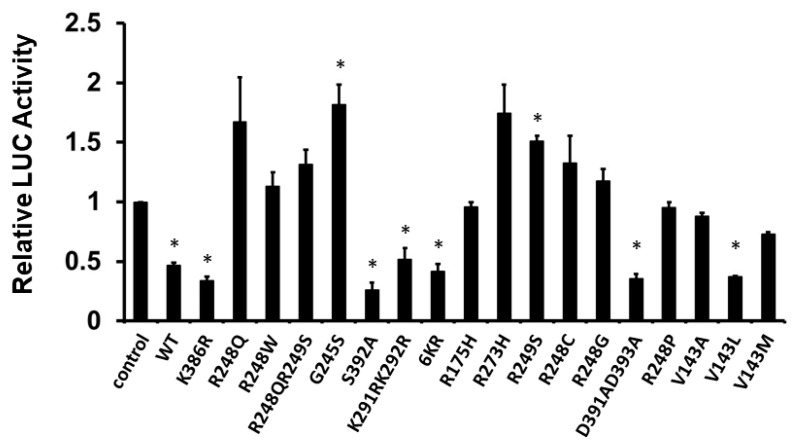
DNA binding domain is required for p53 to down-regulate *PDCD4* transcription. H1299 cells in a 24-well plate were co-transfected with *PDCD4* luciferase reporter plasmid and *pRL-TK Renilla* control vector and various *p53* mutant expression plasmids by Fugene HD. After forty-eight hours of transfection, luciferase activities were analyzed by the Dual-Luciferase Reporter System and normalized to the control *Renilla* activity. Relative LUC activity was calculated and plotted. * indicates a *p*-value < 0.05 compared with the control.

Furthermore, to confirm the result from Figure 3, we next examined the effect of human p53 mutations in the DNA-binding domain and the C-terminal regulatory domain on the transcriptional activity of the *PDCD4* promoter. H1299 cells were co-transfected with the *PDCD4* promoter-LUC reporter plasmid and with either wild-type (WT), mutants of the DNA-binding domain (R248Q, R248W, R248QR249S, G245S, K291RK292R, R175H, R273H, R249S, R248C, R248G, R248P, V143A, V143L, and V143M), or mutants of the C-terminal regulatory domain (K368R, S392A, 6KR, D391AD393A) *p53* expression plasmid. The 6KR mutant represents K370R+K372R+K373R+K381R+K382R+K386R. As shown in Figure 5, in H1299 cells, while the WT p53 and p53 C-terminal regulatory domain mutants repressed *PDCD4* promoter activity, the majority of p53 DNA-binding domain mutants did not reduce *PDCD4* promoter activity, except K291RK292R and V143L, which suggests that the DNA-binding domain is critical for p53-mediated PDCD4 repression.

**Figure 6:**
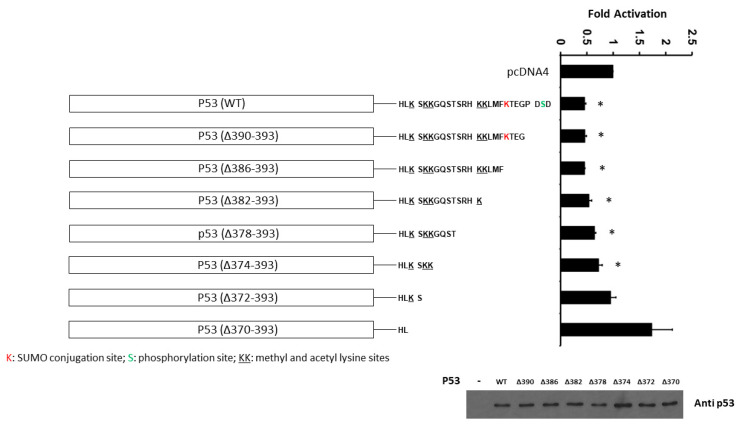
The C-terminal domain (between aa374 and aa370) of p53 is required for p53-mediated PDCD4 repression. H1299 cells in a 24-well plate were co-transfected with *PDCD4* luciferase reporter plasmid and *pRL-TK Renilla* control vector, and various truncated *p53* expression plasmids by Fugene HD. After forty-eight hours of transfection, luciferase activities were analyzed by the Dual-Luciferase Reporter System and normalized to the control *Renilla* activity. Relative LUC activity was calculated and plotted. p53 protein levels in the cells from the reporter assays were confirmed using anti-p53 immunoblotting. * indicates a *p*-value < 0.05 compared with pcDNA4 (control). The original WB can be found in Supplementary Material.

In order to further determine the importance of the C-terminal domain of p53 on PDCD4 promoter activity, we next evaluated the effect of human p53 truncations in the C-terminal regulatory domain on the transcriptional activity of the *PDCD4* promoter. H1299 cells were co-transfected with the *PDCD4* promoter (−242/+355 bp)-LUC reporter plasmid and with either wild-type (WT) or various truncations of the C-terminal domain (Δ390–393, Δ386–393, Δ382–393, Δ378–393, Δ374–393, Δ372–393, and Δ370–393) *p53* expression plasmids. As shown in Figure 6, in H1299 cells, while the WT p53 repressed *PDCD4* promoter activity, the truncations of the p53 C-terminal domain (Δ372–393 and Δ370–393) resulted in the loss of p53-mediated *PDCD4* promoter repression, which suggests that the sequence between aa374 and aa370 of p53 (which contains methylation and acetylation sites) is critical for p53-mediated PDCD4 repression.

## 4. Discussion

Within cells, the network of transcription factors coordinates regulating downstream target genes by responding to a great diversity of pathophysiological stimuli and thus plays a critical role in processing information for cells, including apoptosis/necrosis, cell growth and arrest, developmental control/management, metabolic regulation, pathogenesis, and reproduction [32,36,37,38]. Numerous syndromes and disorders, including autoimmune dysfunctions, cardiovascular diseases, metabolic disorders, cancers, and neurological diseases, can often be caused by mutations of transcription factors. [39,40,41]. The most studied protein and transcription factor in the world, p53, has many functions in apoptosis, senescence, cell cycle regulation, DNA repair, metabolism, redox control, genomic stability, and differentiation [42,43]. The mutations or deletions of the *TP53* gene have been discovered in more than half of human malignancies [1,44]. Herein, we show for the first time that p53 acts as a transcriptional repressor of the human *PDCD4* gene and subsequent PDCD4 protein in human cells.

It is well documented that PDCD4, a tumor suppressor, can suppress tumor proliferation and progression and is often down-regulated in many types of human cancer [45,46,47]. The hallmark of the biochemical function of PDCD4 is as an inhibitor of translation, such as by inhibiting the helicase activity of EIF4A. A previous report has shown that PDCD4 inhibits the translation of p53 mRNA by directly interacting with the 5′-UTR of p53 mRNA [22]. Furthermore, SKP2, an oncogene, promotes breast cancer tumorigenesis and radiation resistance through PDCD4 ubiquitination [48]. Once PDCD4 is ubiquitinated, it is consequently degraded by proteasomes. In the JB6 mouse model, PDCD4 has been shown to inhibit tumor promotion [49]. Moreover, in the PDCD4 knockout animal model, the PDCD4 knockout induces epithelial to mesenchymal transition (EMT) [50]. However, the detailed mechanisms by which PDCD4 behaves as a tumor suppressor are still largely unknown. However, in the present work, we showed that p53 downregulates PDCD4 protein levels and decreases *PDCD4* promoter activity. Our data highlights that *PDCD4* is a novel target gene for the tumor suppressor p53. This information adds to the current knowledge that PDCD4 can be regulated at different levels of epigenetics, transcription, and post-translation, including phosphorylation and miRNA-21 regulation [51].

In addition to its role as an inhibitor of translation, PDCD4 can cooperate with several transcription factors to execute its-related regulation and its role in diseases. For example, PDCD4 can interact with (1) twist1 to inhibit YB-1 expression [33], (2) DAXX to inhibit DAXX-mediated HipK2 function [52], (3) PABP to inhibit translation [53], and (4) p65 to inhibit NF-kB-mediated function [54]. PDCD4 has also been shown to be involved in microglia activation [55], gastric cancer growth [56], apoptosis of placental trophoblasts via the miRNA-21-PDCD4 pathway [57], and cerebral ischemia/reperfusion injury [58]. Since PDCD4 inhibits the translation of p53 mRNA [22], and the current study shows that p53 represses PDCD4 expression, it suggests that p53 and PDCD4 may have a regulatory loop between each other. Such a regulatory loop has been observed in the p53 and MDM2 loop: while p53 up-regulates MDM2 expression, MDM2 facilitates p53 degradation through ubiquitination [59]. However, whether PDCD4 can directly interact with p53 in a two-way feedback loop remains largely unknown. Further, extensive future studies are necessary to analyze this potential regulatory loop and its mechanism.

The post-translational modifications (PTMs) such as acetylation, methylation, phosphorylation, SUMOylation, and ubiquitination influence a wide range of cellular activities, including neurological diseases, metabolism, and cancer development [60,61], especially breast cancer development. In addition, extensive studies demonstrate that human p53 can be modified by PTMs such as phosphorylation (S9, S15, T18, S20, S33, S37, S46, T55, S183, S269, T284, S315, and S392), acetylation (K120, K305, K321, K373, K381, and K382), ubiquitylation (K24, K291, and K292), SUMOylation (K386), and methylation (R333, R335, R337, K370, K372, K373, and K382) [62,63]. The C-terminal regulatory domain of p53 contains several PTM sites, which suggests that p53 is constantly regulated by dynamic PTM regulation. In the present work, we demonstrated that mutations in the DNA-binding domain abolish p53-mediated PDCD4 repression, including R248Q, R248W, R248QR249S, G245S, R175H, R273H, R249S, R248C, R248G, R248P, V143A, and V143M (Figure 5). This result is consistent with previous reports that the DNA-binding domain is critical for p53-mediated transcription [64]. Most mutations in the DNA-binding domain of p53 either disrupt p53’s 3D structure or interfere with p53-DNA contact at promoter regions. Furthermore, many mutations in the DNA-binding domain of p53 are shown in human cancers as “hotspot mutations.” Interestingly, in contrast to the DNA-binding domain, the mutations in the C-terminal regulatory domain of p53 still maintain p53’s ability to repress PDCD4 expression, including K368R, S392A, 6KR, and D391AD393A (Figure 5), which suggests that PTMs play a minor role in p53-mediated PDCD4 repression. 

Additionally, both the N-terminal and C-terminal domains of p53 show an “intrinsic disorder” structure compared to the central regions (including the DNA-binding domain), which show a relatively ordered structure [65]. Using a truncation strategy on the C-terminal regulatory domain of p53, we showed that the region between aa370 and aa374 is required for p53-mediated PDCD4 promoter repression (Figure 6). This result is similar to the previous report [66], which shows that the C-terminal 30 amino acids of p53 are required for sequence-specific binding to p53 response elements. The first explanation is that truncation of the bell-shaped C-terminal domain disrupts p53’s ability to bind to the DNA of the promoter region, even if the C-terminal regulatory domain is relatively disordered in 3D structure. The second explanation is that the C-terminal regulatory domain of p53 has a positive regulatory function in sensing DNA response elements, as mentioned in the previous report [65]. The third possibility is that the sequence between aa374 and aa370 of p53 (which contains methylation and acetylation sites) is specifically essential for p53-mediated PDCD4 repression. However, future studies are necessary to dissect this potential regulatory mechanism in the C-terminal domain of p53.

The PDCD4 protein is heavily post-translationally modified by PTMs, especially phosphorylation (S25, S67, S68, S71, S76, S78, S94, Y152, S313, S317, and S457) [67]. During the current study, we intended to search the potential SUMOylation sites on PDCD4 since SUMOylation is one of the PTMs frequently found on tumor suppressors. However, our results suggest that PDCD4 is not likely to be modified by SUMOylation. 

In conclusion, this study demonstrates that p53 is a novel repressor of the *PDCD4* promoter and that the DNA binding domain and C-terminal regulatory domain (the region between aa370 and aa374) are critical for p53-mediated PDCD4 repression.

## 5. Conclusions

In summary, we show a novel relationship between p53 and PDCD4 for the first time. Our studies suggest that p53 is a novel repressor of the *PDCD4* promoter and that the DNA binding domain and the region of aa370 to aa374 of the C-terminal regulatory domain are critical for p53-mediated PDCD4 repression. Overall, our findings add a new layer of information to our previous understanding of the p53/PDCD4 axis and its functions.

## Data Availability

The data presented in this study are available on request from the corresponding author.

## Supplementary Materials

The following supporting information can be downloaded at: https://www.mdpi.com/article/10.3390/curroncol30020124/s1, Supplementary Material: the original WB blots of figure 1, 2 and 6.
