# Supplementary material for: Tumor Suppressor p53 Down-Regulates Programmed Cell Death Protein 4 (PDCD4) Expression
Source: Curr Oncol. 2023 Jan 27;30(2):1614–25. doi: 10.3390/curroncol30020124 (PMC9955764; doi:10.3390/curroncol30020124)

Figure S1A original images

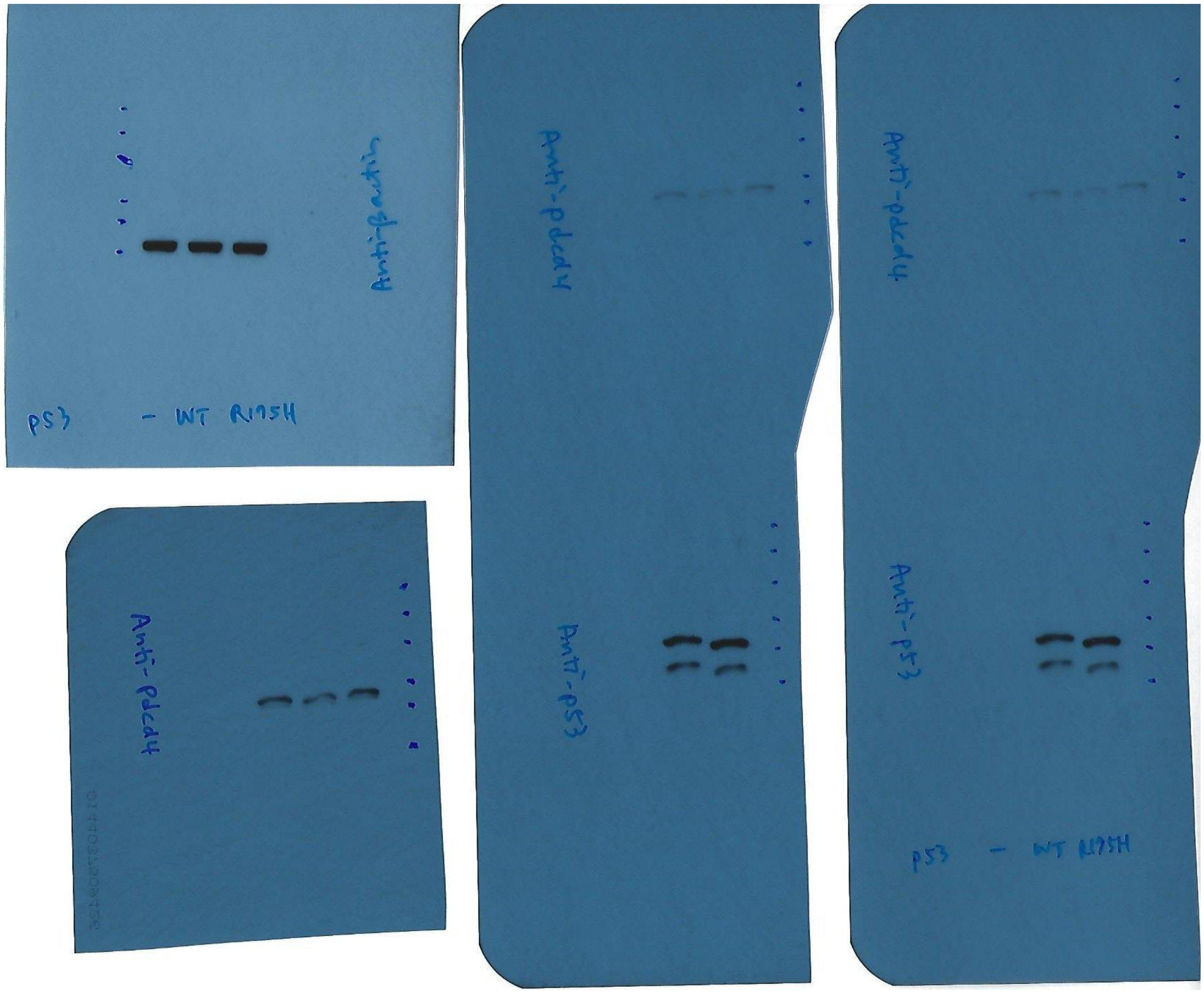

Figure S1B original images

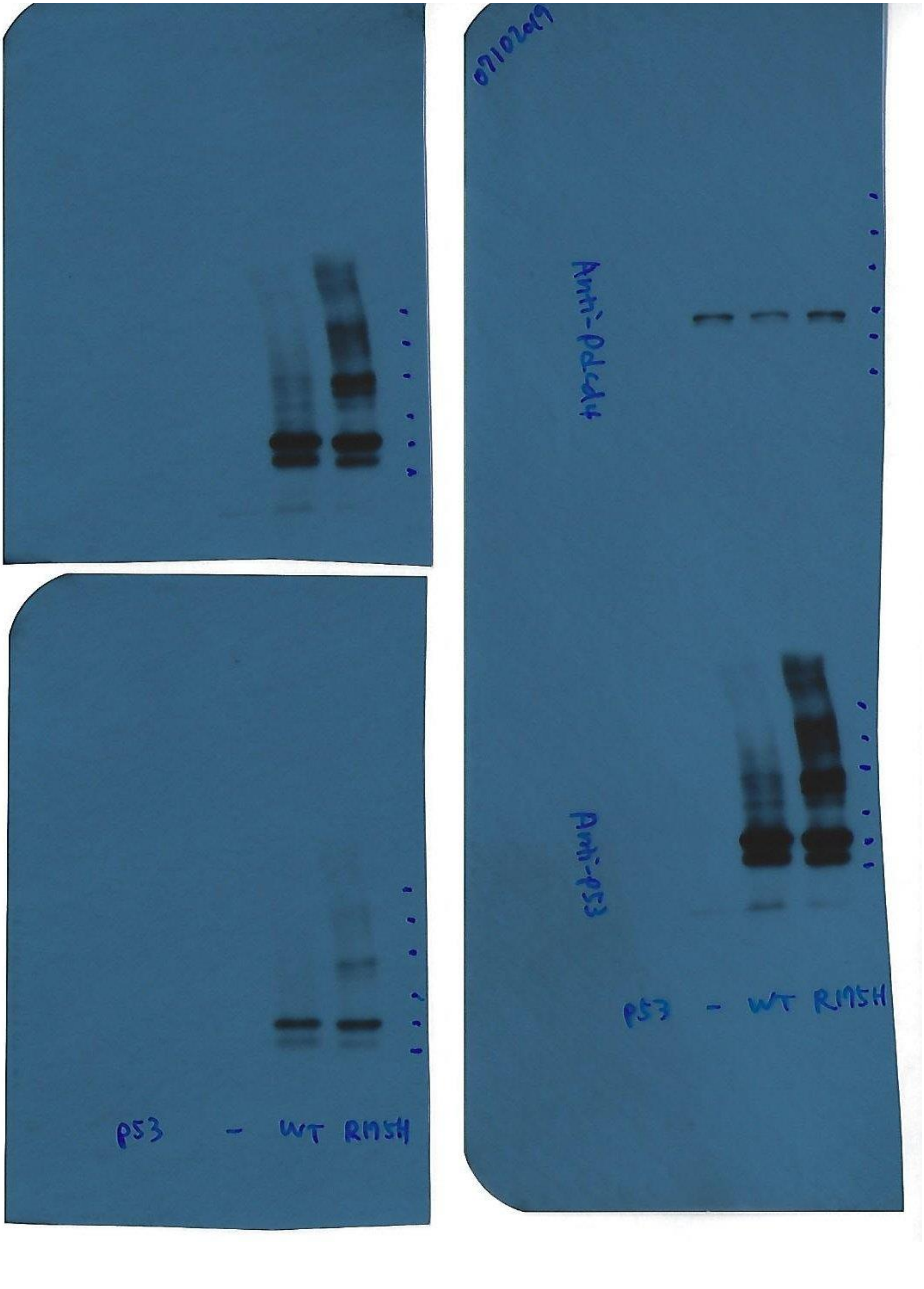

Figure S1C original images

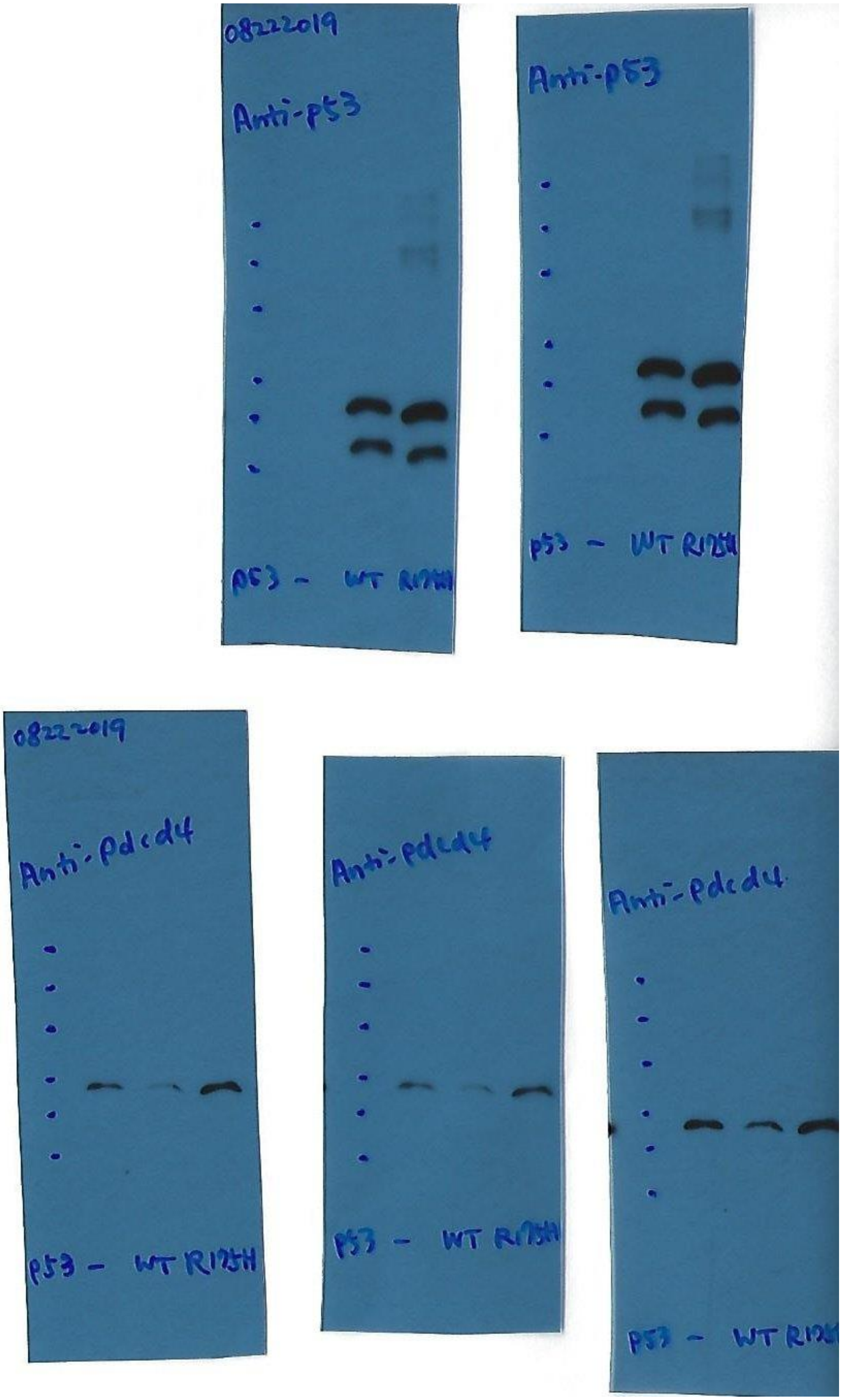

Figure S1D original images

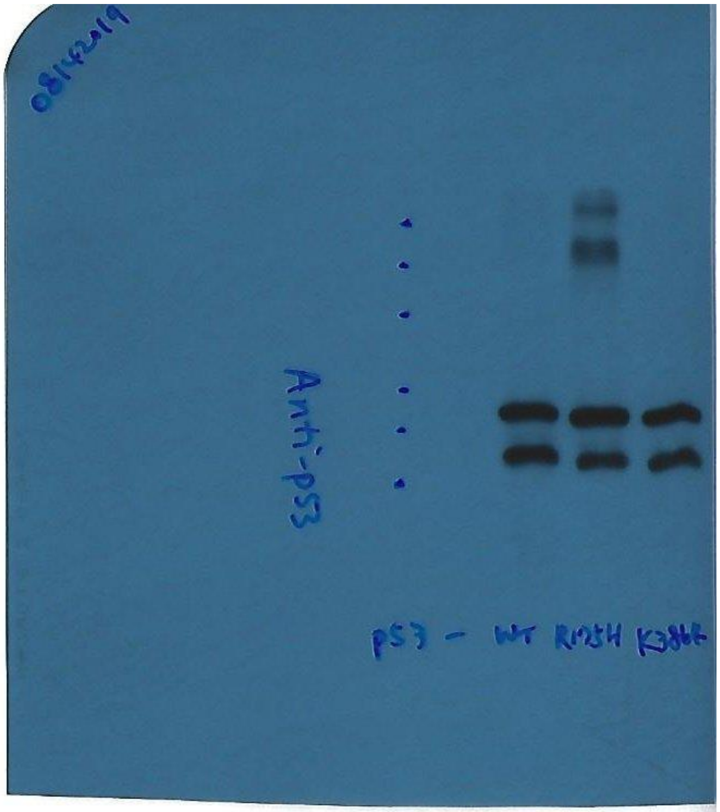

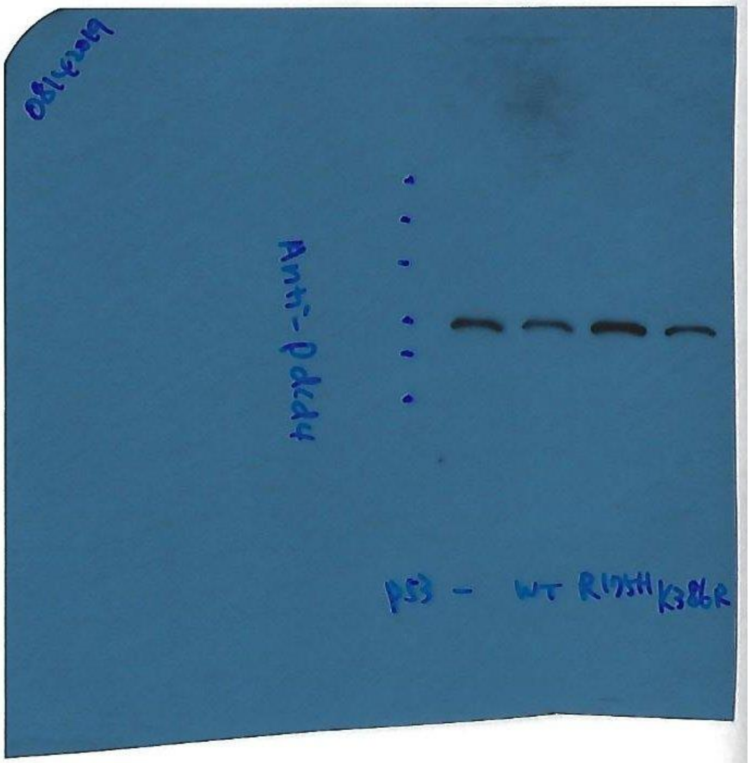

Figure S1D original images

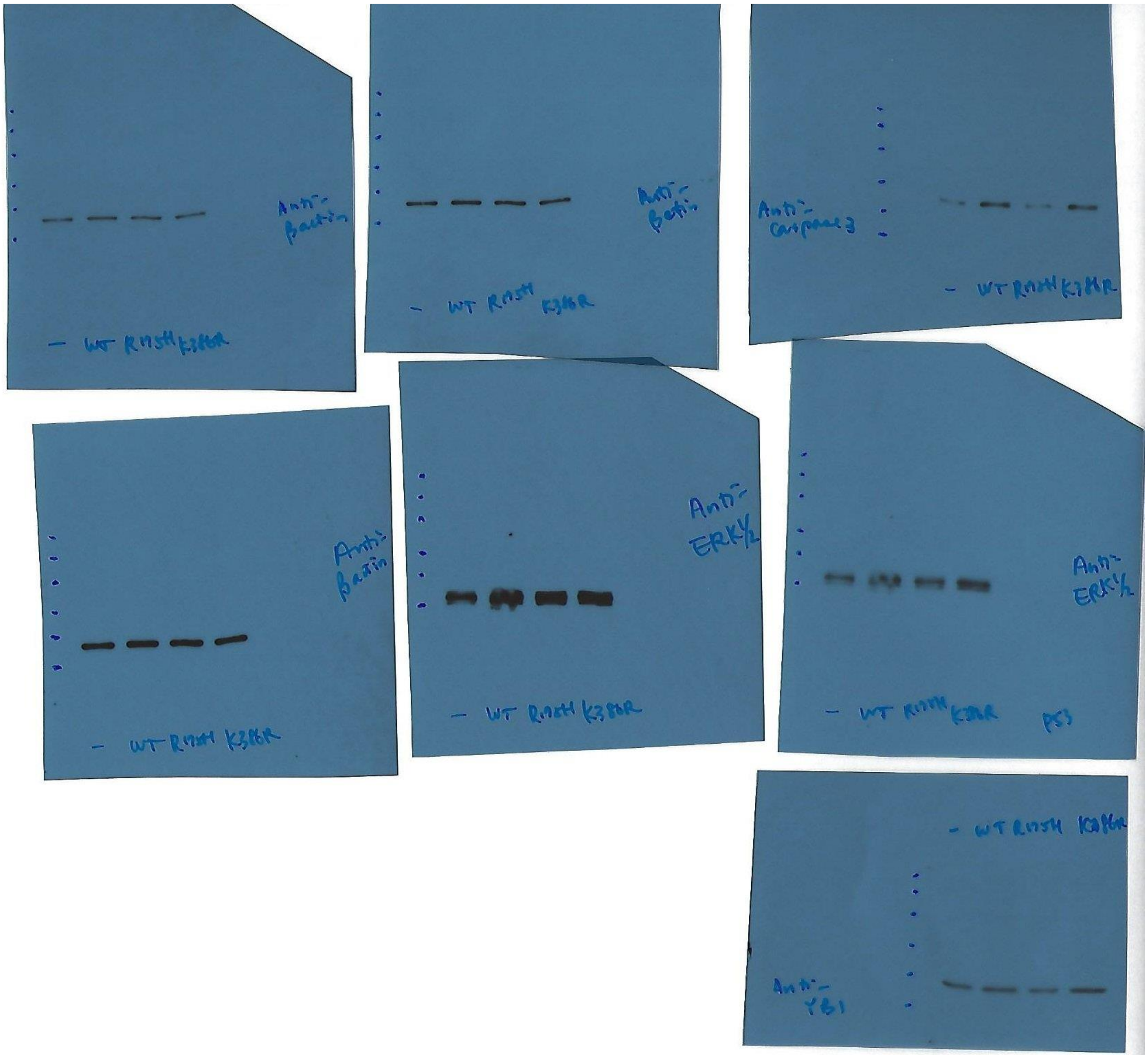

Figure S1E original images

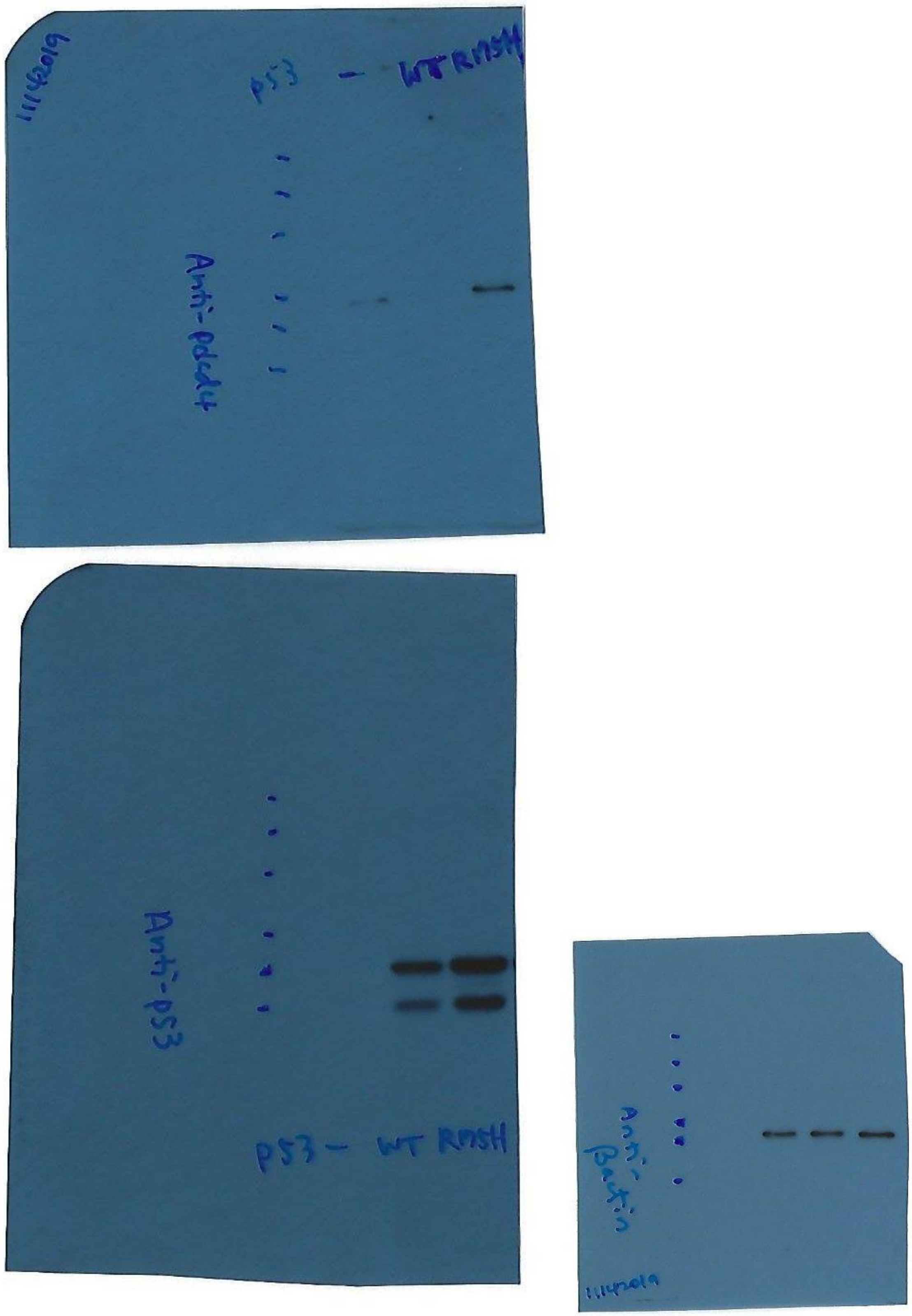

Figure S1F original images

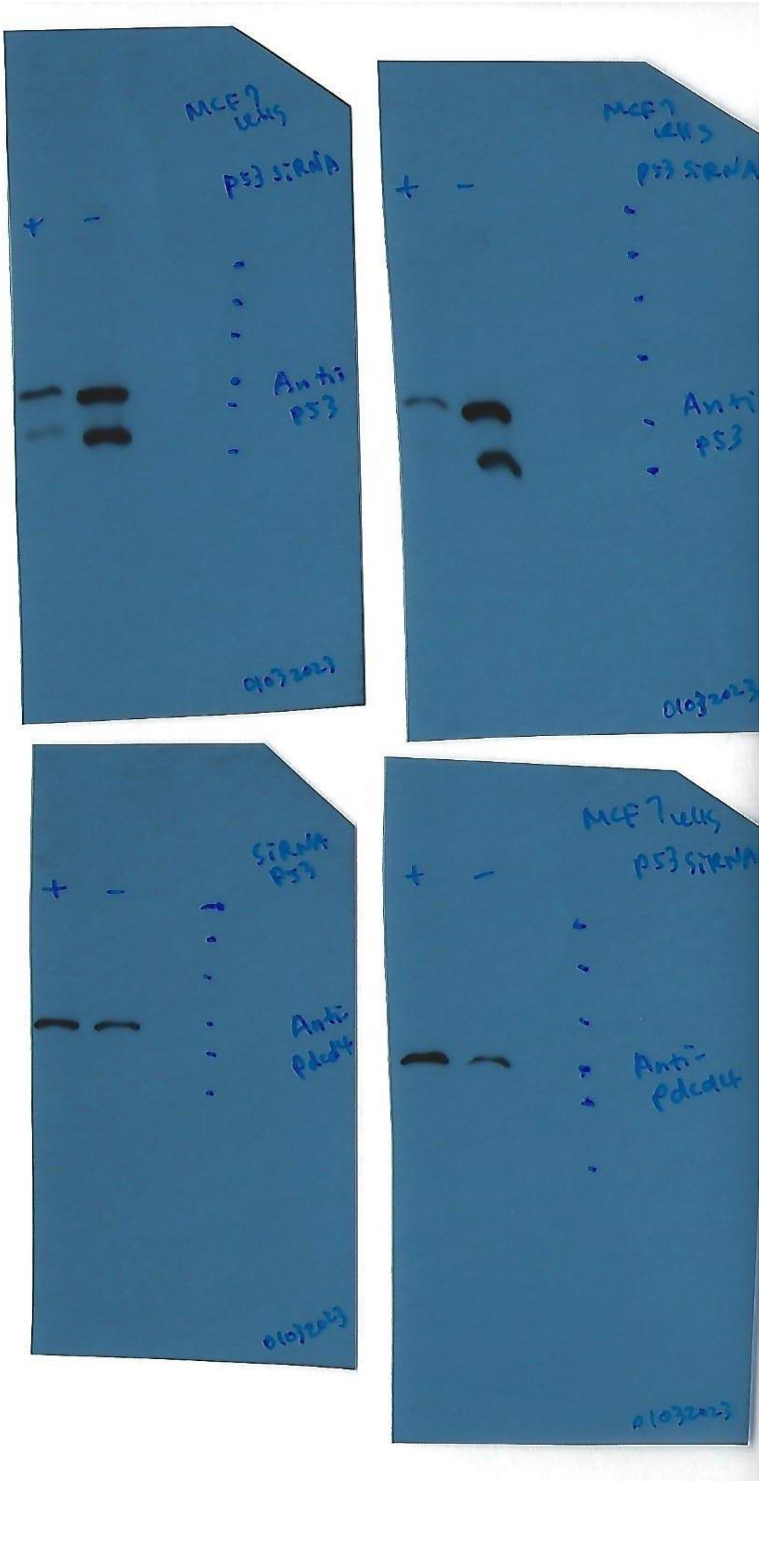

Figure S1F original images

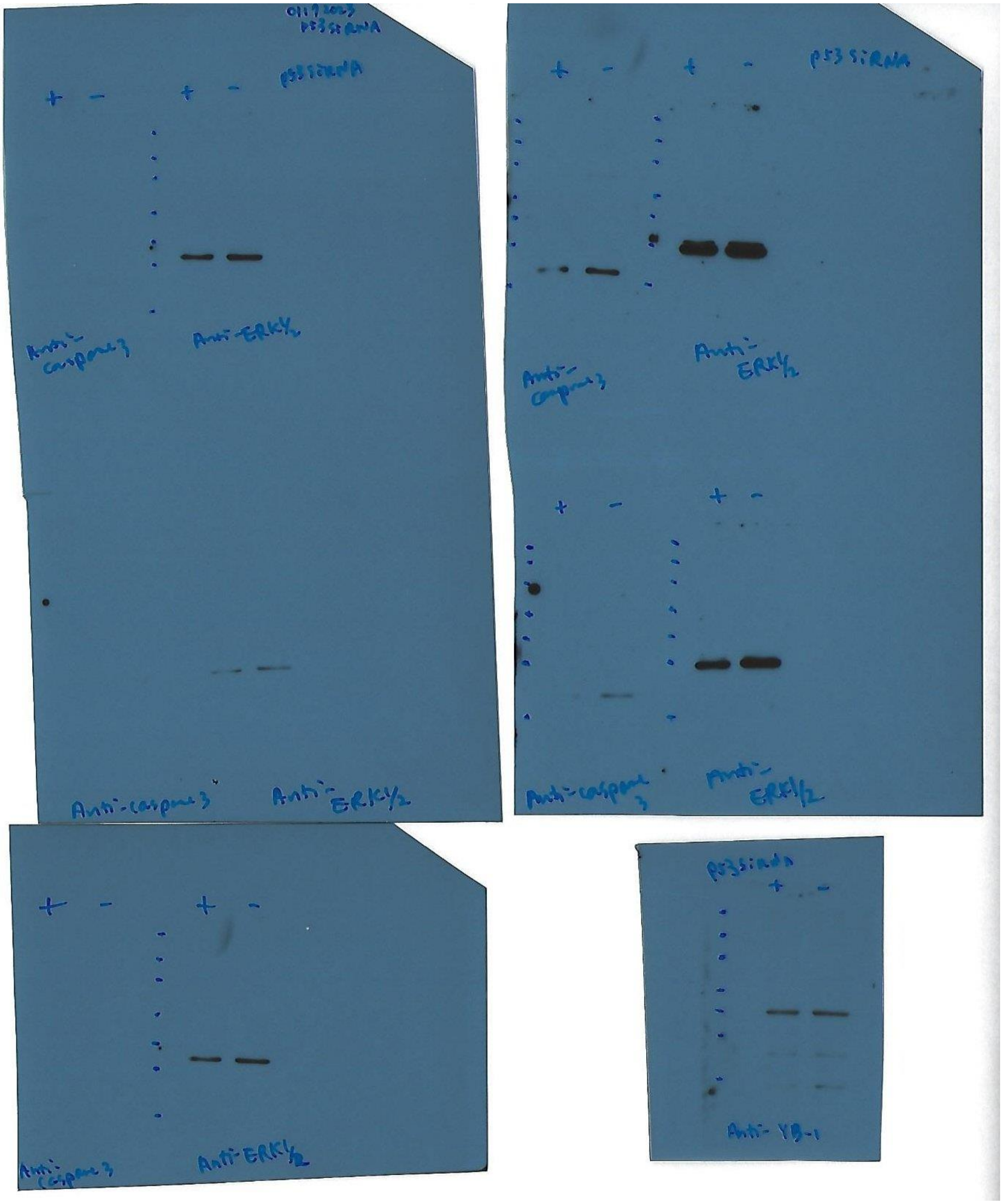

Figure S1F original images

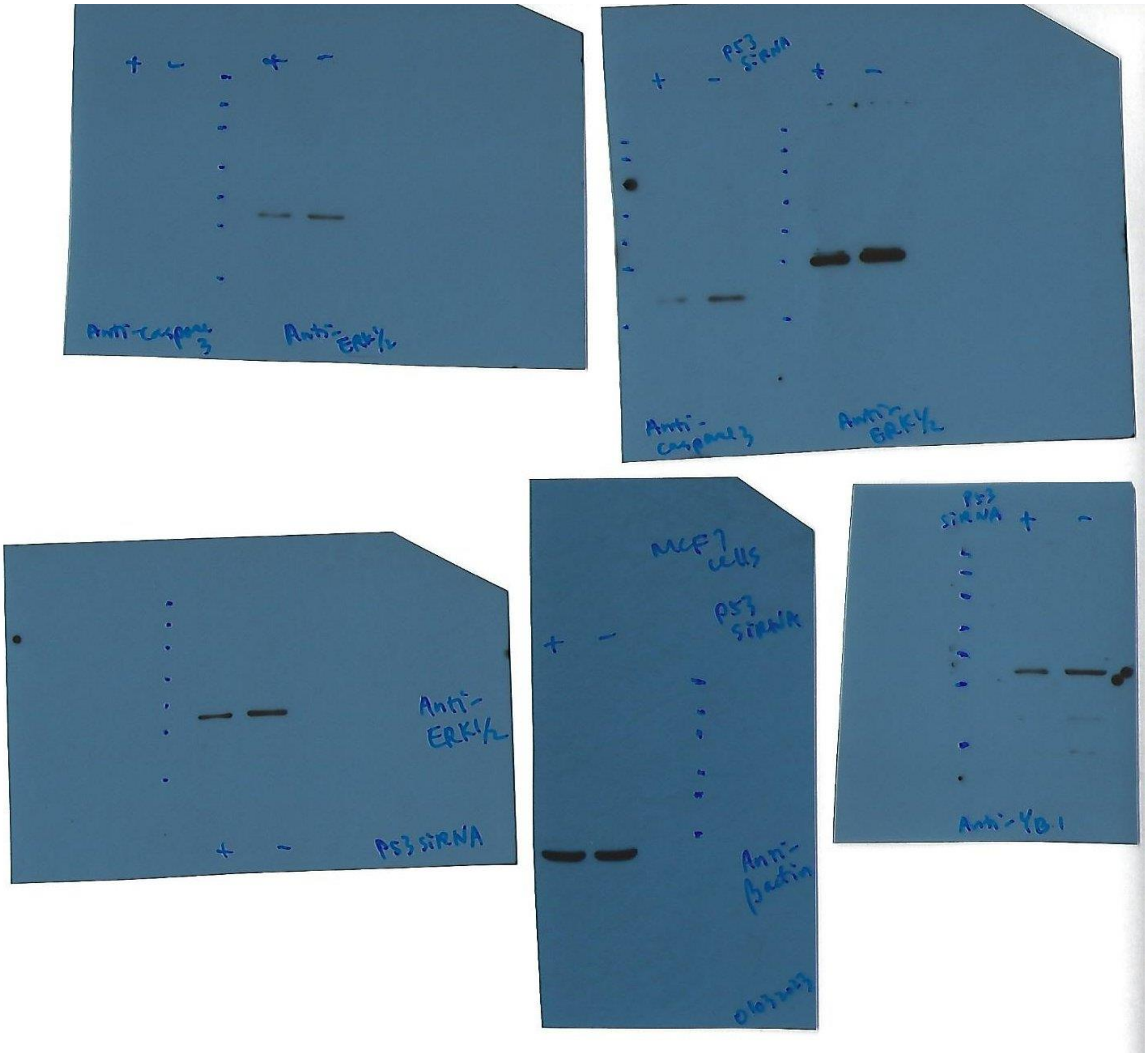

Figure S2 original image

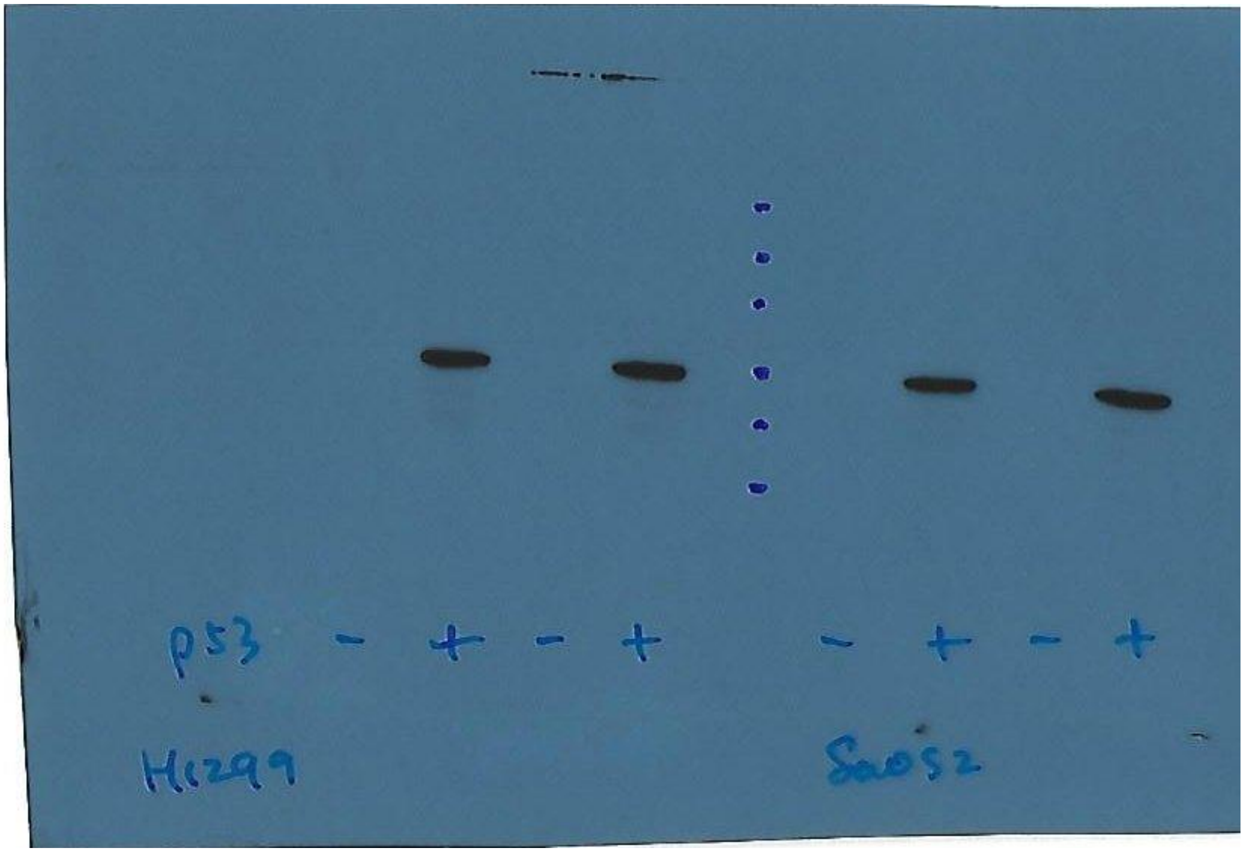

Figure S6 original image

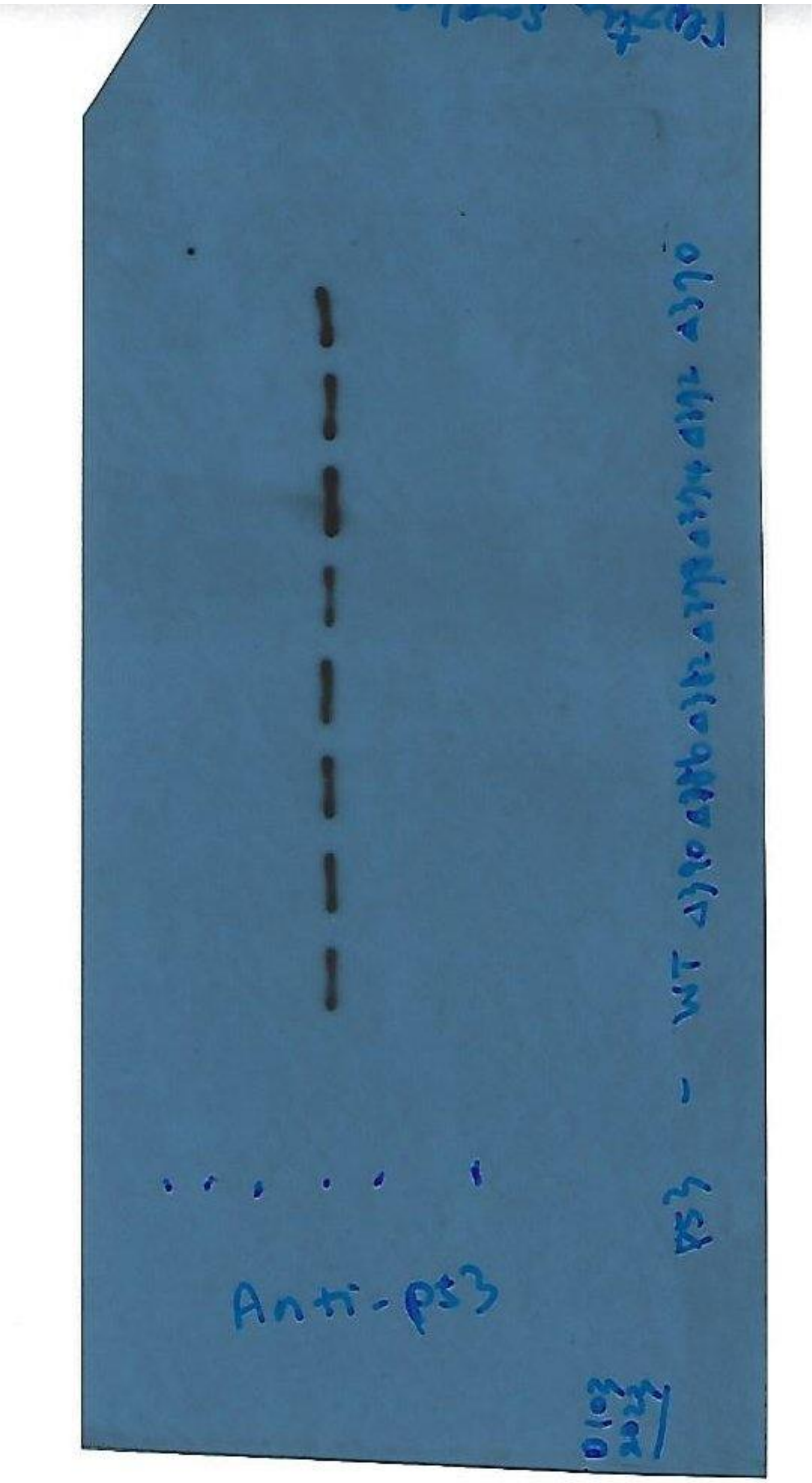

Supplement: Supplementary file 1 [file curroncol-30-00124-s001.zip › curroncol-2120761-supplementary.pdf]
